# BRCA Methylation Testing Identifies a Subset of Ovarian Carcinomas without Germline Variants That Can Benefit from PARP Inhibitor

**DOI:** 10.3390/ijms21249708

**Published:** 2020-12-19

**Authors:** Nora Sahnane, Ileana Carnevali, Giorgio Formenti, Jvan Casarin, Sofia Facchi, Raffaella Bombelli, Eleonora Di Lauro, Domenico Memoli, Annamaria Salvati, Francesca Rizzo, Fausto Sessa, Maria Grazia Tibiletti

**Affiliations:** 1 SC Anatomia Patologica, Ospedale di Circolo, ASST Settelaghi, 21100 Varese, Italy; nora.sahnane@asst-settelaghi.it (N.S.); ileana.carnevali@asst-settelaghi.it (I.C.); eleonora.dilauro@asst-settelaghi.it (E.D.L.); fausto.sessa@uninsubria.it (F.S.); 2 Centro di Ricerca dei Tumori Eredo-Familiari, Dipartimento di Medicina e Chirurgia, University of Insubria, 21100 Varese, Italy; 3 Dipartimento di Ostetricia e Ginecologia, ASST Settelaghi, University of Insubria, 21100 Varese, Italy; giorgio.formenti@asst-settelaghi.it (G.F.); j.casarin@uninsubria.it (J.C.); 4 Dipartimento di Medicina e Chirurgia, University of Insubria, 21100 Varese, Italy; sofia.facchi@uninsubria.it (S.F.); raffaella.bombelli@uninsubria.it (R.B.); 5 Programma di Genomica Medica, AOU SS Giovanni di Dio e Ruggi d’Aragona Università di Salerno, 84131 Salerno, Italy; dmemoli@unisa.it (D.M.); asalvati@unisa.it (A.S.); frizzo@unisa.it (F.R.); 6 Laboratorio di Medicina Molecolare e Genomica Dipartimento di Medicina, Chirurgia e Odontoiatria “Scuola Medica Salernitana” Università di Salerno, Baronissi, 84084 Salerno, Italy

**Keywords:** promoter methylation, *BRCA* genes, PARP inhibitors, ovarian cancer, pyrosequencing

## Abstract

Homologous Recombination Deficiency (HRD) is a frequent feature of high-grade epithelial ovarian carcinoma (EOC), associated with sensitivity to PARP-inhibitors (PARPi). The best characterized causes of HRD in EOCs are germline or somatic mutations in *BRCA1* and *BRCA2* genes. Although promoter methylation is a well-known mechanism of gene transcriptional repression, few data have been published about *BRCA* gene methylation in EOCs. In this retrospective study, we quantitatively analyzed by pyrosequencing a selected series of 90 formalin-fixed (FFPE) primary EOCs without *BRCA* germline mutations. We identified 20/88 (22.7%) EOCs showing *BRCA* promoter methylation, including 17/88 (19.3%) in *BRCA1* and 4/86 (4.6%) in *BRCA2* promoters, one of which showing concomitant *BRCA1* methylation. Mean methylation levels were 49.6% and 45.8% for *BRCA1* and *BRCA2,* respectively, with methylation levels ≥50% in 10/20 methylated EOCs. Constitutive *BRCA* methylation was excluded by testing blood-derived DNA. In conclusion, pyrosequencing methylation analysis of *BRCA* genes is a robust, quantitative and sensitive assay applicable to FFPE samples. Remarkably, a considerable subset of germline *BRCA*-negative EOCs showed somatic methylation and, likely, HRD. A subpopulation of women with *BRCA* methylation, even without *BRCA* mutations, could potentially benefit from PARP-inhibitors; further clinical studies are needed to clarify the predictive role of somatic *BRCA* methylation of PARP-therapy response.

## 1. Introduction

Epithelial ovarian carcinoma (EOC) is the most lethal gynecologic malignancy, and the most frequent cause of cancer-related mortality in women in the world [1]. The average 5-year survival rate is approximately 30% with standard treatments of cytoreductive surgery, and platinum and taxane based chemotherapy [2]. A promising novel therapy for EOC is based on the inhibition of poly(ADP-ribose) polymerase (PARP), which is synthetically lethal in cancer cells with acquired inactivation of the homologous recombination-mediated repair (HR) pathway [2]. Although it is supposed that HR deficiency can arise through germline and somatic mutations of a wider set of homologous recombination repair related genes [3,4,5], the well described causes of HR deficiency in EOC are germline or somatic mutations in the *BRCA1* and *BRCA2* genes that are detected in 12–15% and 5–7% of cases, respectively [6]. Recently, in our country, the Olaparib PARP–inhibitor (PARPi) therapy has been approved (AIFA GU N.140, 17 June 2019) by Health Authorities as single-agent and as maintenance treatment in platinum-sensitive EOC patients with somatic or germline mutations of *BRCA1* and *BRCA2* genes. It is well demonstrated that PARPi therapy improves prognosis in platinum-sensitive EOC patients, particularly in patients with defective homologous recombination mediated repair, especially *BRCA1/2* defects. Although the impact of germline *BRCA* gene deleterious mutations on PARPi and platinum responses in EOC is well established, the clinical relevance of *BRCA* promoter methylation is still unknown [7,8,9,10,11]. It is reported that hypermethylation predominantly occurs for *BRCA1* in 10 to 20% of EOCs, reversely few incidence data regarding *BRCA2* methylation are available [6]. Clinical studies that included screening for HR gene methylation provided conflicting evidence and their accuracy cannot currently be established [7,10,12,13,14]. Of note, the majority of the studies considered methylation data as “all or none” results and some papers have focused on the promoter regions, whose impact on gene transcription has not yet been fully ascertained [15]. Indeed, mainly due to wide concerns regarding the analytic validity of the published studies, the 2020 ESMO recommendation [16] clearly claimed that currently there isn’t enough evidence to determine the clinical validity of *BRCA1* promoter methylation yet, and no datum is available for *BRCA2* gene. The main confounding factors are both of technical and biological types, and are attributable to the measurement of tumor DNA methylation [16].

A new national universal tumor *BRCA1/2* workflow was approved [17] to support treatment choice, however no strategy is available on the proper handling of *BRCA* hypermethylated cases with respect to PARPi therapy.

Although promoter methylation of *BRCA1* and of *BRCA2* gene has not been widely assessed in ovarian cancers, this mechanism is well known to affect other tumor suppressor genes, and, importantly, it is easy to detect in routine diagnostics even when FFPE tumor tissue is the only available material.

Here we analyzed *BRCA1* and *BRCA2* promoter methylation in a series of 90 FFPE EOC, selected for the absence of germline *BRCA1/2* pathogenetic variants, using pyrosequencing analysis to quantitatively detect *BRCA* methylation.

## 2. Results

**Figure 1:**
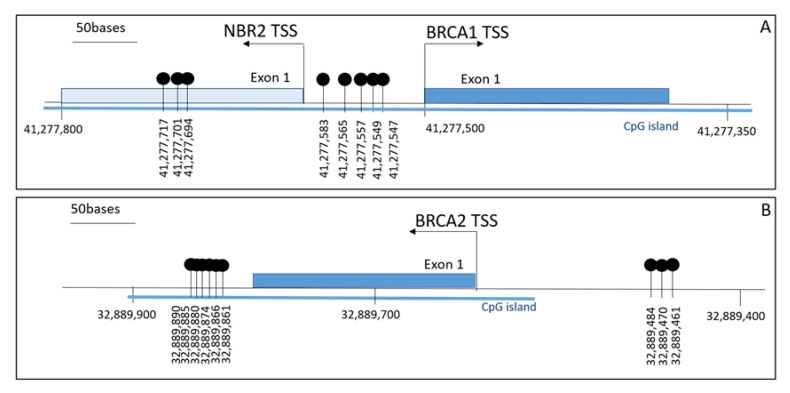
Individual CpG sites investigated in *BRCA1* (**A**) and *BRCA2* (**B**) promoter. Genomic coordinates correspond to the RefSeq NM_007294, transcript variant 1 (*BRCA1* gene) and NM_000059.3 (*BRCA2* gene), using GChr37/hg19 assembly. TSS: Transcription Start Site.

Methylation tests for *BRCA1* and *BRCA2* gene were performed using pyrosequencing. The assay design included two sets of primer for each gene: for *BRCA1* promoter, we selected CpG sites for which a correlation with gene transcription levels was demonstrated [15]. For *BRCA2*, we addressed the regions analyzed by Vos et al. [18], as shown in Figure 1.

Methylation analysis of *BRCA1* and *BRCA2* promoter sequences was performed on 90 primary EOCs, for which it was previously demonstrated the absence of Mismatch Repair (MMR) defects and of pathogenetic germline *BRCA1/2* variants. Pyrosequencing results were obtained for *BRCA1* in 88 out of 90 (97.7%) and for *BRCA2* in 86 out of 90 (95.5%) EOCs. Methylation analyses failed in four cases, which presented poor quality or low amount of tumor DNA.

We identified *BRCA1* promoter hypermethylation in 17 out of 88 (19.3%) EOCs and of BRCA2 methylation in 4 out of 86 (4.6%) EOCs. In one sample, concomitant promoter hypermethylation of both *BRCA1* and *BRCA2* genes was diagnosed (Table S1). Methylation levels ranged between 20.7% and 91.5%, with a mean value of 49.6% and 45.8% for *BRCA1* and *BRCA2*, respectively. On the contrary, 66 out of 88 EOCs showed methylation values of the investigated cytosines that were below the cut-off of 15% and were classified as unmethylated for both genes.

In summary, 20 out of 88 (22.7%) EOCs revealed promoter methylation of *BRCA* genes, 66 cases were classified as unmethylated for both genes, in two cases unmethylated for *BRCA1* promoter, *BRCA2* test failed (Table S1).

No promoter methylation of *BRCA* genes was observed in 9 EOCs of patients carrying germline pathogenetic variants of *BRCA1* and *BRCA2* genes studied for comparison.

All patients affected by methylated EOCs were also studied for constitutive methylation by testing DNA from peripheral blood and no cases of constitutive *BRCA* promoter methylation were observed.

**Figure 2:**
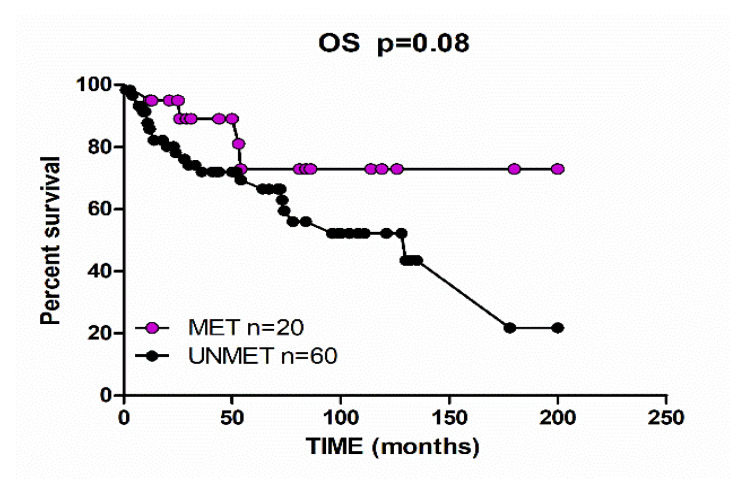
Overall survival at a follow-up time of 200 months, according to *BRCA* gene methylation status.

We compared clinico-pathological features of *BRCA1/2* methylated versus unmethylated EOCs (Table 1), and no statistical significances have been observed between the two different patient populations. Of note, a positive family history for breast or ovarian cancers was observed in 11 out of 68 (16.2%) unmethylated EOCs, and in only 1 out of 20 (5.0%) methylated EOCs. Although no statistical differences between the two groups were observed, it is worth noting that platinum-sensitivity at 6 and 12 months of treatment was recorded, respectively, in 90% and 80% of methylated EOCs, compared to 80% and 72.6% of unmethylated EOCs; time to disease progression was 60 and 52 months for methylated and unmethylated EOCs, respectively. On the contrary, overall survival analyses underlined a consistent better overall survival for the BRCA methylated subset, with a proportion of overall survival at 200 months of 72% compared to 22% in unmethylated EOCs (*p* = 0.08; Figure 2).

## 3. Discussion

In this study, we performed *BRCA1* and *BRCA2* promoter methylation analysis on a selected series of EOCs, all patients were negative for germline pathogenetic variants of *BRCA* genes and no other genes of HR pathway were investigated. To our knowledge, this is the first study performed on selected EOCs without germline *BRCA1* and *BRCA2* mutations. In fact, while the role of deleterious *BRCA* gene mutations on PARPi and platinum responses in EOC is well established, contrasting data have been reported about the impact of *BRCA* gene promoter methylation on the identification of an additional subset of patients that might benefit of PARPi. Above all, in the recent past, several concerns have been raised about the series selection and on technical approaches to measure DNA methylation. In our study, we chose a pyrosequencing technique, as it is a simple and reproducible test to quantitatively detect DNA methylation status at specific genomic loci, and, due to the possibility to analyze small amplicons, it is feasible even on formalin fixed tissues. Moreover, unlike restriction enzyme-based methods, it allows us to potentially address every CpG site of interest, as it is not necessary to have a predefined “consensus” sequence. Importantly, the quantitative data allow us to understand if monoallelic or biallelic methylation is present, or to hypothesize allelic losses associated to allele methylation. Indeed, it has been demonstrated on a cohort of EOC patient-derived xenograft models, that the zygosity of *BRCA1* methylation, along with the number of methylated alleles, is a key determining factor for PARPi sensitivity [19].

The mean methylation levels that we found were 49.6% and 45.8% for *BRCA1* and *BRCA2*, respectively, with methylation levels over 50% in 10 out of 20 methylated EOCs (see Table S1), suggesting the loss of unmethylated alleles, or the presence of constitutional methylation. However, this last mechanism was excluded as we have not found *BRCA* methylation on DNA from peripheral blood of any of the methylated EOC patients. This finding is in agreement with data of Tabano et al. [20], that recently demonstrated that *BRCA* epimutation represent a very rare event in high risk EOC population. The high level of *BRCA1* and *BRCA2* methylation found in this study could be considered to lead to *BRCA* dysfunction due to homozygous promoter methylation.

Previous studies have reported *BRCA* methylation frequencies between 10 and 31% using different approaches [6,19,21,22], namely Methylation-Specific PCR, Methylation Sensitive Restriction endonuclease digest, Methylation Sensitive–MLPA, and, more recently, ddPCR and Genome wide methylation assay [9,19,23]. The majority of papers focused on *BRCA1* gene, while a very small number of data are available on *BRCA2* gene [16].

We identified 20 out of 88 (22.7%) EOCs showing promoter methylation, including 17 out of 88 (19.3%) cases for *BRCA1* and 4 out of 86 (4.6%) for *BRCA2*, one of which showed concomitant methylation of both genes. In agreement with published studies in literature [6] also in this study, *BRCA2* gene is rarely affected by epigenetic silencing and *BRCA1* and *BRCA2* involvement is mutually exclusive. Importantly, our study identified a subset of *BRCA* methylated EOCs comparable in size to the frequency of *BRCA* germline mutated EOCs, and even larger than the group of EOCs with somatic *BRCA* mutations [6,24]. We have confirmed that *BRCA* promoter methylation has never been observed in non-neoplastic ovarian tissue, suggesting that *BRCA* epigenetic silencing is a cancer-specific mechanism, as described by other researchers [21]. Moreover, none of the BRCA germline mutated EOCs harbored promoter methylation, confirming as already reported [16] that this epigenetic mechanism is mutually exclusive with *BRCA* germline variants and does not act as a “second hit” in women carrier of mutations.

Considering ovarian carcinogenesis, we found that *BRCA* promoter methylation does occur in both high and low grade EOCs and in all histological types including serous, endometrioid, and clear cell carcinomas (Table 1) suggesting that this epigenetic mechanism is a common marker of EOC and might be an early event in EOC pathogenesis. As shown in Table 1, the presence of multiple tumors was reported in both methylated and unmethylated EOC patients without significant differences, while breast and/or ovarian cancer positive family history analysis was preferentially recorded in unmethylated EOC patients. Even if the difference did not reach statistical significance due to the limited population, it suggests that *BRCA* methylation pattern preferentially characterizes sporadic EOCs. With regard to clinical features, it is noteworthy that the majority of methylated EOCs demonstrated platinum-sensitivity at 6 and 12 months (Table 1) of treatments. Interestingly, the methylated EOC patients revealed a strong and consistent better overall survival rate, compared to the unmethylated patients (*p* = 0.08; Figure 2). This favorable outcome strongly reminds the survival rates reported for EOC associated with *BRCA* germline mutation versus non BRCA-mutated EOC [9,25,26], supporting the idea that *BRCA* methylated EOCs could be a new subset of cancers with impaired *BRCA* function.

Although a limit of our study is the absence of a RNA-level analysis, data from other authors reported that hypermethylation of *BRCA1* promoter reduces gene expression [15,23,27] and robust data from a recent large meta-analysis [15] remarkably demonstrated that methylation of the specific promoter sequences investigated in our work produces *BRCA1* and *BRCA2* transcriptional down-regulation. In addition, another paper has found that EOCs with *BRCA1* promoter hypermethylation lost *BRCA1* immunohistochemical expression, consistently with the gene silencing [21].

In summary, this study demonstrated, by pyrosequencing approach, that 22.7% of EOC women without germline deleterious *BRCA* variants showed *BRCA* promoter methylation. Given the high proportion of platinum-sensitive cases, and the clinical outcome similar to that of germline *BRCA*-mutated EOCs, it is reasonable to suppose that this represents an additional subset of HR-deficient EOC that could benefit from PARPi therapy. New evidences suggest significative efficacy of PARPi therapy on tumors with *BRCA* promoter methylation. Swisher et al. [14] has reported up to 50% of response rate for Rucaparib. Currently, due to lack of evidence, neither established guideline nor therapeutic options are available in patients with the above described tumor features.

In conclusion, our study revealed that pyrosequencing analysis of *BRCA1* and *BRCA2* gene promoter is a robust and sensitive assay for BRCA promoter methylation assessment in FFPE samples of EOCs. Promoter hypermethylation has been demonstrated in a consistent subset of non-familial EOCs showing different histological types and a high proportion of platinum-sensitive cases.

Our data support the hypothesis that *BRCA* promoter methylation plays an important role in the pathogenesis of EOCs without germline mutations of these genes and that the detection of such epigenetic event by a quantitative pyrosequencing approach allows to identify a sizeable subset of EOCs that could benefit from PARP-inhibitor therapy.

## 4. Materials and Methods

### 4.1. Patients and Samples

This is a retrospective monocentric study using samples from FFPE archival tissues of EOCs carefully selected among patients referred for genetic counselling and genetic predisposition testing to Cancer Genetic Service of ASST Settelaghi in Varese from 2008 to 2019. All patient data were previously reported by Carnevali et al. [28]. In detail, EOC samples were selected for absence of MMR defects and absence of germline BRCA1 or BRCA2 pathogenic variants. No other genes of HR pathway were investigated.

All 90 primary EOCs have been surgically removed before chemotherapy treatment and were evaluated by two independent gyneco-pathologists (F.S. and E.D.L.) EOC were classified according to 2014 WHO [29], and tumor histology was stratified into high-grade (74) and low grade (14) EOCs. EOCs showed different histological types, namely, 63 were serous, 22 endometrioid, and 6 clear cells carcinomas. The mean age at diagnosis was 59.5 (of 33–75 years). Clinical data including the presence of multiple tumors, family history, and platinum-sensitivity were available for 88 patients; time to progression and overall survival data were available for 80 patients.

Tumor DNA was extracted from three representative sections of EOCs. The percentages of neoplastic cells of the samples ranged from 40 to 95%, with 84% of samples showing a tumor area content greater or equal to 60%. As a control group, 10 non-neoplastic ovarian samples were selected from surgical specimens of women carrying pathogenetic variants of *BRCA1* or *BRCA2* gene that underwent salpingo-ovariectomy for prophylactic purpose. In addition, 9 EOCs from women carrier of *BRCA1* or *BRCA2* pathogenetic variant were analyzed for comparison.

This study was conducted according to the principles of the Helsinki Declaration and was approved (12 March 2019) by Research Ethics Committee of Insubria (ID 238 of 2018). Written informed consent was obtained from each participant.

### 4.2. BRCA1 and BRCA2 Gene Promoter Methylation Test

About 100–200 ng of genomic DNA was bisulfite-converted with the EZ DNA Methylation Kit (Zymo Research, 17062 Murphy Ave. Irvine, CA, USA.). *BRCA1* and *BRCA2* promoter methylation was determined by pyrosequencing (QIAGEN, Hilden, Germany) across 8 CpG dinucleotides within the *BRCA1* and 9 CpG whitin *BRCA2* promoter. The core promoter of *BRCA1* encompasses the non-coding exon 1 and part of intron 1 of this gene and of exon 1 and part of intron 1 of the neighboring gene *NBR2*, as annotated by USCS database (chr17: 41,276,000–41,279,000, GChr37/hg19 assembly). The 8 CpG dinucleotides fall within the non-coding exon 1 of *BRCA1* (chr17: 41,277,595–41,277,289). The screened *BRCA2* region encompasses a 500-nucleotide sequence (chr13:32,889,461–32,889,890) in the gene promoter (Figure 1). Primers and PCR conditions of pyrosequencing analysis are reported in Table S2. First, to set-up the methylation tests we analyzed artificial control samples at different percentages of DNA methylation (0, 10, 50, and 100%) by appropriately mixing commercial fully methylated DNA and fully unmethylated DNA (Human WGA Methylated and Non-methylated DNA Set, Zymo Research 17062 Murphy Ave. Irvine, CA, USA). Data from three independent amplification and pyrosequencing experiments of these four samples demonstrated that methylation tests are able to quantify the presence of methylated cytosines with a good linearity (Figure S1). To determine the limit of Blank (LoB), i.e., the highest analyte concentration expected to be found when replicates of a blank sample containing no analyte are tested, we analyzed commercial fully unmethylated samples in 10 different runs and a subset of 10 non neoplastic ovarian tissues as “negative controls”. We set the LoB for *BRCA1* and *BRCA2* methylation tests at a value of 10%, corresponding to the mean value plus three standard deviations of 10 independent measures (Table S3). Subsequently, to set the limit of detection (LoD), we analyzed, for each primer set, data from three independent pyrosequencing analyses of the 10%-methylated control. The obtained values ranged from 6.94 to 14% of methylation, thus we set the LoD at a cut-off of 15% for both genes.

## Figures and Tables

**Table 1:** Clinico-pathological data in relationship to methylation status of ovarian cancers.

	Methylated EOCs (20)		Unmethylated EOCs (68)	*p*-Value
	*BRCA1*	*BRCA2*		
N. of cases	17 *	3 *	68	
Age (mean, years)	56.9	63.6	58.7	0.8
	(60.25)			
Grade				
High	18		56	0.5
Low	2		12	
Histological types				
Serous	13	2	45	0.6
Endometrioid	4	-	16	
Clear cell	-	1	3	
Others	-	-	4	
Multiple tumors	4 **	-	7 ***	0.26
FH	1		11	0.28
Platinum sensitivity				
6 months	18/20 (90%)		50/62 (80%)	0.5
12 months	16/20 (80%)		45/62 (72.6%)	0.57
Time to progression (median, months)	60		52	0.68

Legend: FH (Family History): presence of family history of breast and ovarian cancers, *: one patient had ovarian cancers *BRCA1* and *BRCA2* methylated, ** 3 breasts, 1 endometrial, 1 kidney, *** 3 breasts, 1 colorectal, 1 endometrial cancers.

## Supplementary Materials

Supplementary materials can be found at https://www.mdpi.com/1422-0067/21/24/9708/s1.

## Abbreviations

EOC	Epithelial ovarian carcinoma
PARP	poly(ADP-ribose) polymerase
PARPi	poly(ADP-ribose) polymerase inhibitor
AIFA	Agenzia Italiana del Farmaco
FFPE	Formalin-fixed paraffin embedded tissue
MMR	MisMatch Repair system
