# Supplementary material for: BRCA Methylation Testing Identifies a Subset of Ovarian Carcinomas without Germline Variants That Can Benefit from PARP Inhibitor
Source: Int J Mol Sci. 2020 Dec 19;21(24):9708. doi: 10.3390/ijms21249708 (PMC7767143; doi:10.3390/ijms21249708)
Supplement: Supplementary file 1 [file ijms-21-09708-s001.pdf]

International Journal of
Molecular Sciences

MDPI

Supplementary Table S 1. *BRCA1* and *BRCA2* promoter methylation by pyrosequencing

ID number	BRCA1 (% met)	U/M	BRCA2 (% met)	U/M
1	6.53	U	4.37	U
2	10.8	U	8.5	U
3	6.93	U	2.7	U
4	0	U	0	U
5	**65.4**	**M**	4.9	U
6	2.4	U	1.53	U
7	5	U	7.17	U
8	6.3	U	0	U
9	4.33	U	4.57	U
10	8.23	U	3.83	U
11	3.87	U	4	U
12	7.6	U	7.57	U
13	3.1	U	4.23	U
14	4.73	U	2.57	U
15	0	U	3.73	U
16	3.83	U	3.63	U
17	0	U	6.4	U
18	**80.23**	**M**	0	U
19	4.43	U	3.03	U
20	**91.47**	**M**	3.9	U
21	4.4	U	3.27	U
22	3.6	U	5.23	U
23	**58.53**	**M**	2.5	U
24	4.1	U	5.27	U
25	**30.63**	**M**	3.57	U
26	9.77	U	4	U
27	**42.2**	**M**	3.2	U
28	5.23	U	4.32	U
29	**55.47**	**M**	10	U
30	**23.68**	**M**	4.28	U
31	10	U	5.3	U
32	**34.03**	**M**	4	U
33	5.8	U	4	U
34	**52.37**	**M**	4.33	U
35	0	U	0	U
36	5.97	U	3	U
37	0	U	0	U
38	0	U	ne	ne
39	6.47	U	4.2	U
40	**35.87**	**M**	5.73	U
41	5.97	U	5.13	U
42	9.23	U	0	U
43	6.1	U	5.2	U
44	5.8	U	4.73	U
45	5.9	U	3.77	U
ID number	BRCA1 (% met)	U/M	BRCA2 (% met)	U/M

46	8	U	3.57	U
47	0	U	0	U
48	14	U	3.4	U
49	4.2	U	**34.31**	**M**
50	5.8	U	10	U
51	3.77	U	3.3	U
52	7.33	U	**58.3**	**M**
53	6.43	U	0	U
54	**31.07**	**M**	0	U
55	7.5	U	10	U
56	**20.73**	**M**	ne	ne
57	**64.77**	**M**	4.1	U
58	0	U	**44.07**	**M**
59	6.1	U	5.6	U
60	5.47	U	3.4	U
61	ne	ne	ne	ne
62	12	U	U	u
63	11	U	7.1	U
64	6.53	U	3.33	U
65	8.73	U	0	U
66	**65.63**	**M**	4.13	U
67	7.23	U	6.13	U
68	5.33	U	3.67	U
69	4.2	U	0	U
70	5.73	U	3.13	U
71	9.73	U	10.16	U
72	4.3	U	9.7	U
73	0	U	U	U
74	**42.07**	**M**	**46.6**	**M**
75	8	U	4.83	U
76	3.57	U	3.27	U
77	4.6	U	4.27	U
78	0	U	3.57	U
79	4.33	U	3.4	U
80	ne	ne	ne	ne
81	4.83	U	4.43	U
82	**49.47**	**M**	2.77	U
83	3.37	U	5.37	U
84	6.5	U	2.4	U
85	8	U	6.57	U
86	6.83	U	2.9	U
87	10	U	10	U
88	0	U	3.33	U
89	7.43	U	2.87	U
90	5.33	U	2.23	U

Legend. M, methylated; U, unmethylated; ne, not evaluable. Percentages represent the average of all methylation values at Individual CpG sites investigated in BRCA1 and BRCA2 promoter. Genomic coordinates of all CpG sites are specified in Figure 1. All analyses have been performed in duplicate

International Journal of
Molecular Sciences
MDPI

Supplementary Table S2. Primers and PCR conditions of pyrosequencing analysis.

Gene		Primers	AT (°C)	Amplicon lenght (bp)
BRCA1	M3-F	AGGTTAGAGGGTAGGTATTTTATGG	56	105bp
	M3-R^{biotin}	ACTCTAAATTAACCACCCAATCT		
	M3-Seq	ATGGTAAATTTAGGTAGAATTTTT		
	M4-F	ATTTAGTATTTTGAGAGGTTGTTGTT	56	150bp
	M4-R^{biotin}	AAAACCCCACAACCTATCC		
	M4-Seq	TTTGAGAGGTTGTTGTTTA		
BRCA2	M1-F	GTTAGTGGTGGTGGTAGTG	56	106bp
	M1-R^{biotin}	AACCTTTCTCTCAAACATAAATAAAAATTA		
	M1-Seq	GTTTTTAGTAGTTTTAGTTTAG		
	M4-F	GGAGTAGTTGTGGTATTGT	TD (61-58)	149bp
	M4-R^{biotin}	TCACAAATCTATCCCCTCAC		
	M4-Seq	GGAGTAGTTGTGGTATTGTT		

Legend: F. forward primer; R, reverse primer; Seq, sequencing primer; AT, annealing temperature; bp, base pair; TD, touch down

Supplementary Table S3. Inter-experiments variability and definition of the limit of blank , LoB

Test	Unmethylated samples mean±SD	Unmethylated samples mean+3SD	Methylated samples (mean±SD)
BRCA1 MET3	0.73%±1.41	4.96%	91.23%±6.74
BRCA1 MET4	0.5%±0.98	3.44%	76.3%±8. 4
BRCA2 MET1	2.31%±2.59	**10%**	83.53%±7
BRCA2 MET4	0.4%±1.13	3.8%	95.22%±3.35

Fully unmethylated samples were tested in 10 different runs and the results were recorded to perform LoB calculation (LoB is the highest analyte concentration expected to be found when replicates of a blank sample containing no analyte are tested). We set the Lob for all methylation tests at a value of 10%, corresponding to the mean value plus three standard deviations of 10 independent measures.

International Journal of *Molecular Sciences*

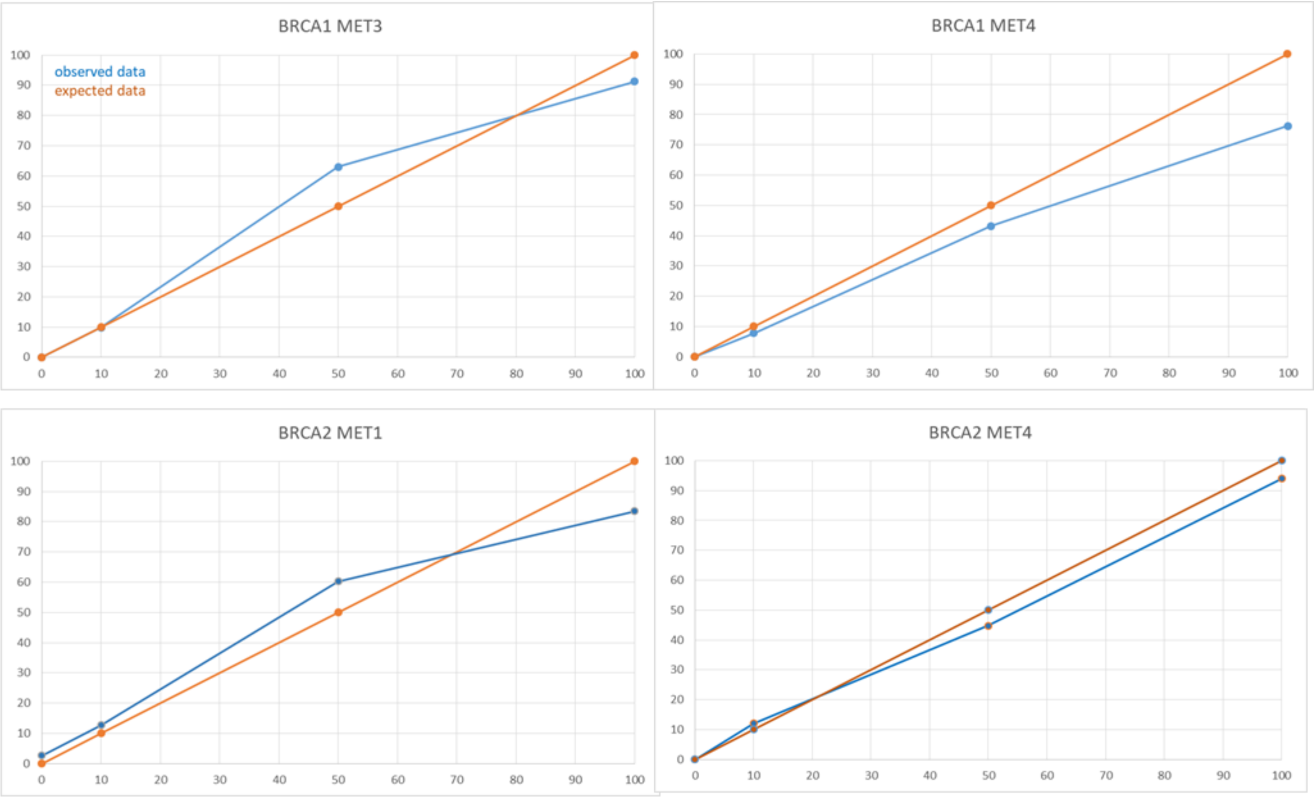

Supplementary Figure S1. To set-up the methylation tests we analyzed artificial control samples at different percentages of DNA methylation (0%, 10%, 50%, 100%) by appropriately mixing commercial fully methylated DNA and fully unmethylated DNA (Human WGA Methylated & Non-methylated DNA Set, Zymo Research). Data from three independent amplification and pyrosequencing experiments of these four samples demonstrated that methylation tests are able to quantify the presence of methylated cytosines with a good linearity. Two assays for each gene have been set up. On X- and Y-axes methylation levels (%) are reported.
